# Community confounding in joint species distribution models

**DOI:** 10.1038/s41598-022-15694-6

**Published:** 2022-07-18

**Authors:** Justin J. Van Ee, Jacob S. Ivan, Mevin B. Hooten

**Affiliations:** 1 Department of Statistics, Colorado State University, Fort Collins, 80523 USA; 2 Colorado Parks and Wildlife, Fort Collins, 80526 USA; 3 Department of Statistics and Data Sciences, The University of Texas at Austin, Austin, 78712 USA

**Keywords:** Ecology, Mathematics and computing

## Abstract

Joint species distribution models have become ubiquitous for studying species-environment relationships and dependence among species. Accounting for community structure often improves predictive power, but can also affect inference on species-environment relationships. Specifically, some parameterizations of joint species distribution models allow interspecies dependence and environmental effects to explain the same sources of variability in species distributions, a phenomenon we call community confounding. We present a method for measuring community confounding and show how to orthogonalize the environmental and random species effects in suite of joint species distribution models. In a simulation study, we show that community confounding can lead to computational difficulties and that orthogonalizing the environmental and random species effects can alleviate these difficulties. We also discuss the inferential implications of community confounding and orthogonalizing the environmental and random species effects in a case study of mammalian responses to the Colorado bark beetle epidemic in the subalpine forest by comparing the outputs from occupancy models that treat species independently or account for interspecies dependence. We illustrate how joint species distribution models that restrict the random species effects to be orthogonal to the fixed effects can have computational benefits and still recover the inference provided by an unrestricted joint species distribution model.

## Introduction

Historically, species distributions have been modeled independently from each other due to unavailability of multispecies datasets and computational restraints. However, ecological datasets that provide insights about collections of organisms have become prevalent over the last decade thanks to efforts like Long Term Ecological Research Network (LTER), National Ecological Observatory Network (NEON), and citizen science surveys^1^. In addition, technology has improved our ability to fit modern statistical models to these datasets that account for both species environmental preferences and interspecies dependence. These advancements have allowed for the development of joint species distribution models (JSDM)^2–4^ that can model dependence among species simultaneously with environmental drivers of occurrence and/or abundance.

Species distributions are shaped by both interspecies dynamics and environmental preferences^5–8^. JSDMs integrate both sources of variability and adjust uncertainty to reflect that multiple confounded factors can contribute to similar patterns in species distributions. Some have proposed that JSDMs not only account for biotic interactions but also correct estimates of association between species distributions and environmental drivers^3,9^, while others claim JSDMs cannot disentangle the roles of interspecies dependence and environmental drivers^5^. We address why JSDMs can provide inference distinct from their concomitant independent SDMs, how certain parameterizations of a JSDM induce confounding between the environmental and random species effects, and when deconfounding these effects may be appealing for computation and interpretation.

Because of the prevalence of occupancy data for biomonitoring in ecology, we focus our discussion of community confounding in JSDMs on occupancy models, although we also consider a JSDM for species density data in the simulation study. The individual species occupancy model was first formulated by MacKenzie et al.^10^ and has several joint species extensions^4,11–16^. We chose to investigate the impacts of community confounding on the probit model since it has been widely used in the analysis of occupancy data^4,13,17^. We also developed a joint species extension to the Royle-Nichols model^18^ and consider community confounding in that model.

We use the probit and Royle-Nichols occupancy models to improve our understanding of montaine mammal communities in what follows. We show that including unstructured random species effects in either occupancy model induces confounding between the fixed environmental and random species effects. We demonstrate how to orthogonalize these effects in the model and compare the resulting inference compared to models where species are treated independently.

Unlike previous approaches that have applied restricted regression techniques similar to ours, we use it in the context of well-known ecological models for species occupancy and intensity. While such approaches have been discussed in spatial statistics and environmental science, they have not been adopted in settings involving the multivariate analysis of community data. We draw parallels between restricted spatial regression and restricted JSDMs but also highlight where the methods differ in goals and outcomes. We find that the computational benefits conferred by performing restricted spatial regression also hold for some joint species distribution models.

### Royle-Nichols joint species distribution model

We present a JSDM extension to the Royle-Nichols model^18^. The Royle-Nichols model accounts for heterogeneity in detection induced by the species’ latent intensity, a surrogate related to true species abundance. Abundance, density, and occupancy estimation often requires an explicit spatial region that is closed to emmigration and immigration. In our model, the unobservable intensity variable helps us explain heterogeneity in the frequencies we observe a species at different sites without making assumptions about population closure. In the “Model” section, we further discuss the distinctions between abundance and intensity in the Royle-Nichols model.

The Royle-Nichols model utilizes occupancy survey data but provides inference distinct from the basic occupancy model^10^. In the Royle-Nichols model, we estimate individual detection probability for homogeneous members of the population, whereas in an occupancy model, we estimate probability of observing at least one member of the population given that the site is occupied. Furthermore, the Royle-Nichols model allows us to relate environmental covariates to the latent intensity associated with a species at a site, while in an occupancy model, environmental covariates are associated with the species latent probability of occupancy at a site. Species intensity and occupancy may be governed by different mechanisms, and inference from an intensity model can be distinct from that provided by an occupancy model^19–21^. Cingolani et al.^20^ proposed that, in plant communities, certain environmental filters preclude species from occupying a site and an additional set of filters may regulate if a species can flourish. Hence, certain covariates that were unimportant in an occupancy model may improve predictive power in an intensity model.

### Community confounding

Species distributions are shaped by environment as well as competition and mutualism within the community^8,22,23^. Community confounding occurs when species distributions are explained by a convolution of environmental and interspecies effects and can lead to inferential differences between a joint and single species distribution model as well as create difficulties for fitting JSDMs. Former studies have incorporated interspecies dependence into an occupancy model^4,11–16^, and others have addressed spatial confounding^1,17,24,25^, but none of these explicitly addressed community confounding. However, all Bayesian joint occupancy models naturally attenuate the effects of community confounding due to the prior on the regression coefficients. The prior, assuming it is proper, induces regularization on the regression coefficients^26^ that can lessen the inferential and computational impacts of confounding^27^. Furthermore, latent factor models like that described by Tobler et al.^4^ restrict the dimensionality of the random species effect which should also reduce confounding with the environmental effects.

We address community confounding by formulating a version of our model that orthogonalizes the environmental effects and random species effects. Orthogonalizing the fixed and random effects is common practice in spatial statistics and often referred to as restricted spatial regression^27–31^. Restricted regression has been applied to spatial generalized linear mixed models (SGLMM) for observations $\varvec{y},$ which can be expressed as

1 $$\begin{aligned} \varvec{y}&\sim [\varvec{y}|\varvec{\mu }, \varvec{\psi }], \end{aligned}$$

2 $$\begin{aligned} g(\varvec{\mu })&= \varvec{X}\varvec{\beta } + \varvec{\eta }, \end{aligned}$$

3 $$\begin{aligned} \varvec{\eta }&\sim \mathcal {N}(\varvec{0}, \varvec{\Sigma }), \end{aligned}$$

where $g(\cdot )$ is a link function, $\varvec{\psi }$ are additional parameters for the data model, and $\varvec{\Sigma }$ is the covariance matrix of the spatial random effect. In the SGLMM, prior information facilitates the estimation of $\varvec{\eta },$ which would not be estimable otherwise due to its shared column space with $\varvec{\beta }$^30^. This is analogous to applying a ridge penalty to $\varvec{\eta },$ which stabilizes the likelihood. Another method for fitting the confounded SGLMM is to specify a restricted version:

4 $$\begin{aligned} \varvec{y}&\sim [\varvec{y}|\varvec{\mu }, \varvec{\psi }], \end{aligned}$$

5 $$\begin{aligned} g(\varvec{\mu })&= \varvec{X}\varvec{\delta } + (\varvec{I}-\varvec{P}_{\varvec{X}})\varvec{\eta }, \end{aligned}$$

6 $$\begin{aligned} \varvec{\eta }&\sim \mathcal {N}(\varvec{0}, \varvec{\Sigma }), \end{aligned}$$

where $\varvec{P}_{\varvec{X}}=\varvec{X}(\varvec{X}\varvec{X})^{-1}\varvec{X}'$ is the projection matrix onto the column space of $\varvec{X}.$ In the unrestricted SGLMM, the regression coefficients $\varvec{\beta }$ and random effect $\varvec{\eta }$ in (1) compete to explain variability in the latent mean $\varvec{\mu }$ in the direction of $\varvec{X}$^27^. In the restricted model, however, all variability in the direction of $\varvec{X}$ is explained solely by the regression coefficients $\varvec{\delta }$ in (4)^31^, and $\varvec{\eta }$ explains residual variation that is orthogonal to $\varvec{X}$. We refer to $\varvec{\beta }$ as the conditional effects because they depend on $\varvec{\eta }$, and $\varvec{\delta }$ as the unconditional effects.

Restricted regression, as specified in (4), was proposed by Reich et al.^28^. Reich et al.^28^ described a disease-mapping example in which the inclusion of a spatial random effect rendered one covariate effect unimportant that was important in the non-spatial model. Spatial maps indicated an association between the covariate and response, making inference from the spatial model appear untenable. Reich et al.^28^ proposed restricted spatial regression as a method for recovering the posterior expectations of the non-spatial model and shrinking the posterior variances which tend to be inflated for the unrestricted SGLMM.

Several modifications of restricted spatial regression have been proposed^30,32–35^. All restricted spatial regression methods seek to provide posterior means $\text {E}\left( \delta _j|\varvec{y}\right)$ and marginal posterior variances $\text {Var}\left( \delta _j|\varvec{y}\right)$, $j=1,...,p$ that satisfy the following two conditions^36^:

1. $$\text {E}\left( \varvec{\delta }|\varvec{y}\right) = \text {E}\left( \varvec{\beta }_{\text {NS}}|\varvec{y}\right)$$ and,
2. $$\text {Var}\left( \beta _{\text {NS,}j}|\varvec{y}\right) \le \text {Var}\left( \delta _{j}|\varvec{y}\right) \le \text {Var}\left( \beta _{\text {Spatial,}j}|\varvec{y}\right)$$ for $$j=1,...,p$$,

where $\varvec{\beta }_{NS}$ and $\varvec{\beta }_{Spatial}$ are the regression coefficients corresponding to the non-spatial and unrestricted spatial models, respectively.

The inferential impacts of spatial confounding on the regression coefficients has been debated. Hodges and Reich^29^ outlined five viewpoints on spatial confounding and restricted regression in the literature and refuted the two following views:

1. Adding the random effect $\varvec{\eta }$ corrects for bias in $\varvec{\beta }$ resulting from missing covariates.
2. Estimates of $\varvec{\beta }$ in a SGLMM are shrunk by the random effect and hence conservative.

The random effect $\varvec{\eta }$ can increase or decrease the magnitude of $\varvec{\beta }$, and the change may be galvanized by mechanisms not related to missing covariates. Therefore, we cannot assume the regression coefficients in the SGLMM will exceed those of the restricted model, nor should we regard the estimates in either model as biased due to misspecification. Confounding in the SGLMM causes $\text {Var}\left( \beta _j|\varvec{y}\right) \ge \text {Var}\left( \delta _j|\varvec{y}\right)$, $j=1,...,p$, because of the shared column space of the fixed and random effects. Thus, we refer to the conditional coefficients as conservative with regard to their credible intervals, not their posterior expectations.

Reich et al.^28^ argued that restricted spatial regression should always be applied because the spatial random effect is generally added to improve predictions and/or correct the fixed effect variance estimate. While it may be inappropriate to orthogonalize a set of fixed effects in an ordinary linear model, orthogonalizing the fixed and random effect in a spatial model is permissible because the random effect is generally not of inferential interest. Paciorek^37^ provided the alternative perspective that, if confounding exists, it is inappropriate to attribute all contested variability in $\varvec{y}$ to the fixed effects. Hanks et al.^31^ discussed factors for deciding between the unrestricted and restricted SGLMM on a continuous spatial support. The restricted SGLMM leads to improved computational stability, but the unconditional effects are less conservative under model misspecification and more prone to type-S errors: The Bayesian analogue of Type I error. Fitting the unrestricted SGLMM when the fixed and random effects are truly orthogonal does not introduce bias, but it will increase the fixed effect variance. Given these considerations, Hanks et al.^31^ suggested a hybrid approach where the conditional effects, $\varvec{\beta }$, are extracted from the restricted SGLMM. This is possible because the restricted SGLMM is a reparameterization of the unrestricted SGLMM. This hybrid approach leads to improved computational stability but yields the more conservative parameter estimates. We describe how to implement this hybrid approach for joint species distribution models in the “Community confounding” section.

Restricted regression has also been applied in time series applications. Dominici et al.^38^ debiased estimates of fixed effects confounded by time using restricted smoothing splines. Without the temporal random effect, Dominici et al.^38^ asserted all temporal variation in the response would be wrongly attributed to temporally correlated fixed effects. Houseman et al.^39^ used restricted regression to ensure identifiability of a nonparametric temporal effect and highlighted certain covariate effects that were more evident in the restricted model (i.e., the unconditional effects’ magnitude was greater). Furthermore, restricted regression is implicit in restricted maximum likelihood estimation (REML). REML is often employed for debiasing the estimate of the variance of $\varvec{y}$ in linear regression and fitting linear mixed models that are not estimable in their unrestricted format^40^. Because REML is generally applied in the context of variance and covariance estimation, considerations regarding the effects of REML on inference for the fixed effects are lacking in the literature.

In ecological science, JSDMs often include an unstructured random effect like $\varvec{\eta }$ in (1) to account for interspecies dependence, and hence can also experience community confounding between $\varvec{X}$ and $\varvec{\eta }$ analogous to spatial confounding. Unlike a spatial or temporal random effect, we consider random species effects to be inferentially important, rather than a tool solely for improving predictions or catch-all for missing covariates. An orthogonalization approach in a JSDM attributes contested variation between the fixed effects (environmental information) and random effect (community information) to the fixed effect.

We describe how to orthogonalize the fixed and random species effects in a suite of JSDMs and present a method for detecting community confounding. In the simulation study, we test the efficacy of our method for detecting confounding, show that community confounding can lead to computational difficulties similar to those caused by spatial confounding^31^, and highlight that, for some models, restricted regression can improve model fitting. We also investigate the inferential implications of community confouding and restricted regression in JSDMs by comparing outputs from the SDM, unrestricted JSDM, and restricted JSDM of the Royle-Nichols and probit occupancy models fit to mammalian camera trap data. Lastly, we discuss other inferential and computational methods for confounded models and consider their appropriateness for joint species distribution modeling.

## Model

### Data model

The probit and Royle-Nichols occupancy models were developed for analyzing multispecies binary detection data, $y_{ijk}$, arising from a zero-inflated Bernoulli process with probability of success $p_{ijk}$, where $i=1,\dots ,n$, $j=1,\dots ,J_i$, and $k=1,\dots ,K$ correspond to sites, occasions, and species, respectively. Occupancy data of this form have traditionally been analyzed in a latent variable framework^10,41,42^. In what follows, we let $z_{ik} \sim \text {Bern}(\psi _{ik})$ be an indicator on whether species *k* occupies site *i*. Given a site is occupied, we detect species *k* on occasion *j* with some probability $p_{ijk}$, such that $(y_{ijk}|z_{ik}=1)\sim \text {Bern}(p_{ijk})$, but if species *k* is absent from the site, we have zero probability of detecting it, $P(y_{ijk}=0|z_{ik}=0)=1$.

The probit occupancy model is so named because it links $\psi _{ik}$ and $p_{ijk}$ to occupancy and detection covariates $\varvec{x}_{ik}$ and $\varvec{w}_{ijk}$, respectively, with the standard normal CDF $\Phi$. The probit link function can be paired with data augmentation^17,43–45^ to yield efficient Gibbs samplers for the occupancy and detection regression coefficients $\varvec{\beta }$ and $\varvec{\alpha }$, respectively.

Royle and Nichols^18^ introduced a method for analyzing occupancy data that explicitly modeled the probability of detecting species *k* at a site as a function of a surrogate related to the true species abundance. Assuming there are $N_{ik}$ individuals of species *k* in sample region *i* and that all individuals in species *k* on the sample unit have identical detection probabilities and are detected independently of other individuals, the probability of detecting at least one of these individuals can be expressed as

7 $$\begin{aligned} \rho _{ijk} = 1-(1-r_{jk})^{N_{ik}}, \end{aligned}$$

where $r_{jk}$ is a binomial sampling probability that a particular individual of species *k* is detected on occasion *j*. While the Royle-Nichols model facilitates inference on number of individuals of species *k*, $N_{ik}$, at each site when all the assumptions are met, we do not interpret them as such because sites are not necessarily closed in camera trap studies due to mobile species with home ranges larger than the sampling radius of the camera. Note that $\rho _{ijk}$ in (7) corresponds to the species probability of detection conditional on an intensity process. This is distinct from $p_{ijk}$ in the probit model that is conditional on an occupancy process.

The nonlinear function of $r_{jk}$ and $N_{ik}$ in (7) involves more parameters than would be identifiable in a typical occupancy model, especially when the individual detection probability is heterogeneous across occasions (e.g., $r_{jk}$ are heterogeneous). In the heterogeneous case, $r_{jk}$ is connected to covariates with the logit link function:

8 $$\begin{aligned} \text {logit}(r_{jk})=f(\varvec{w}_{jk}, \varvec{\alpha }_k), \end{aligned}$$

where $f(\varvec{w}_{ijk}, \varvec{\alpha }_k)$ is a linear function of the detection covariates $\varvec{w}_{ijk}$ and regression parameters $\varvec{\alpha }_k$.

### Modeling interspecies dependence

We extend both occupancy models to account for interspecies dependence by including random species effects in their process models. Following Royle and Nichols^18^, we assume $N_{ik}\sim \text {Pois}(\lambda _{ik})$, where $\lambda _{ik}$ is mean intensity of species *k* at site *i*. We let $\varvec{\lambda }$ denote the vector of site specific intensities stacked across the *K* species in the community. To model interspecies dependence, we specify the conditional multivariate normal distribution:

9 $$\begin{aligned} \text {log}(\varvec{\lambda })&\sim \mathcal {N}(\varvec{X}\varvec{\beta } + \varvec{\eta }, \varvec{\Sigma _{\lambda }})\text {, } \end{aligned}$$

10 $$\begin{aligned} \varvec{\eta }&\sim \mathcal {N}(\varvec{0}, \varvec{\Sigma }_{spp}\otimes \varvec{I}_n), \end{aligned}$$

where $\varvec{X}$ is a block-diagonal matrix of the *K* species design matrices, $\varvec{\beta } = (\varvec{\beta }'_1, \dots , \varvec{\beta }'_K)'$ is a stacked vector of species specific regression coefficients, $\varvec{\eta }$ represents the random species effects, and $\varvec{\Sigma }_{spp}$ is a species covariance matrix, and $\varvec{\Sigma _{\lambda }}$ is a matrix that allows for additional covariance structures such as spatial dependence. For our purposes of comparing the SDM, unrestricted JSDM, and restricted JSDM for differences galvanized by community confounding, we assumed a simple independent structure for $\text {log}(\varvec{\lambda })$ and set $\varvec{\Sigma _{\lambda }}=\tau \varvec{I}$.

In the probit model, we include a random species effect in the latent probability of occupancy: $\Phi (\varvec{\psi })=\varvec{X}\varvec{\beta }+\varvec{\eta }$, where $\varvec{\psi }$ is a vector of site specific occupancy probabilities stacked across the *K* species in the community and $\varvec{X}$, $\varvec{\beta }=(\varvec{\beta }'_1,\dots ,\varvec{\beta }'_K)'$, and $\varvec{\eta }$ are defined as above.

In both occupancy models, $\varvec{\eta }$ allows for dependence between all *K* species in the community at each site. In the probit model, $\varvec{\eta }$ characterizes interspecies dependence in the probability of occupancy, whereas in the Royle-Nichols model interspecies dependence is characterized in the species latent intensities. Just as certain environmental features may not preclude species occupancy but can curb intensity, some species may coexist in a region but not be able to jointly flourish^46^. Hence, interspecies dependence on latent intensity is conceptually distinct from interspecies dependence on probability of occupancy and may lead to inferential differences in $\varvec{\eta }$ in the two occupancy models.

Tobler et al.^4^ developed a joint occupancy model that accounts for community structure using a latent variable approach. They express the latent probability of occupancy of species *k* at site *i* as

11 $$\begin{aligned} \Phi (\psi _{ik})=\varvec{x}'_{i}\varvec{\beta }_{k}+\varvec{l}'_i\varvec{\theta }_k, \end{aligned}$$

where $\varvec{l}'_i$ is a vector of length *T* of latent variables, and $\varvec{\theta }_k$ are species specific regression coefficients. The latent variable model (LVM) is a computationally efficient and implicitly accounts for community structure. Other occupancy models have included interspecies dependence in the structure of the regression coefficients. Known as multispecies models, these models assume the species specific regression coefficients $\varvec{\beta }_k$ stem from a common multivariate normal distribution $\varvec{\beta }_k\sim \mathcal {N}(\varvec{\mu }, \varvec{\Sigma _{\beta }})$ where $\varvec{\mu }$ is the typical response of a species to covariates $\varvec{x}$ and $\varvec{\Sigma _{\beta }}$ allows for dependence in different species response to the same covariates^47^. In our study of mammalian camera trap data, each species is modeled with unique covariates, and we do not consider shared environmental responses.

Scheffe^48^ stipulated that the levels of a random effect are draws from a population, and the draws are not of interest in themselves but only as samples from the larger population, which is of interest. In more recent literature, the term random effect is used more broadly. Hodges and Clayton^49^ categorized modern definitions of a random effect into three different varieties. The definition commonly used in spatial statistics is, the levels of the effect arise from a meaningful population, but they are the whole population and these particular levels are of interest. We adopt this definition for the random species effects in (9). In practice, some levels of the population will likely not be included in the random species effects. For example, in Ivan et al.^50^, cameras were baited and arranged to capture all members of the mammalian community, but several species were excluded from the random species effects due to a lack of detections.

### Priors

We specified normal priors for the regression coefficients, $\varvec{\beta }$, in the intensity and occupancy processes of the Royle-Nichols and probit models, respectively to facilitate comparison with the occupancy and spatial confounding literature. We also specified normal priors for the detection coefficients, $\varvec{\alpha }$, in the observation model and the conjugate Inverse-Wishart prior for the species covariance matrix $\varvec{\Sigma }_{spp}$. A more general alternative to the Inverse-Wishart prior is to apply a Cholesky decomposition, $\varvec{\Sigma }_{spp} = \varvec{L}\varvec{D}^{-1}\varvec{L}'$, where $\varvec{L}$ is lower diagonal with ones along the diagonal and $\varvec{D}$ is diagonal with positive diagonal elements, and specify priors for the lower diagonal elements of $\varvec{L}$ and diagonal elements of $\varvec{D}$^51^. We found the Inverse-Wishart prior suitable for our inferential goals, but see Chan and Jeliazkov^51^ for alternative covariance matrix priors.

The joint posterior distribution associated with our model is

12 $$\begin{aligned} \begin{aligned}{}&[\varvec{\alpha }, \varvec{\beta }, \varvec{\lambda }, \varvec{N}, \varvec{\Sigma }_{spp}| \varvec{y}] \propto \\&\prod _{k=1}^{K}{\left( \prod _{i=1}^{n}{\left( \prod _{j=1}^{J_i}{\bigg ( [y_{ijk}|N_{ik},\varvec{\alpha }_k] \bigg ) }[N_{ik}|\lambda _{ik}]\right) }[\varvec{\alpha }_k][\varvec{\beta }_k]\right) }[\varvec{\lambda }|\varvec{\beta }_1, \cdots , \varvec{\beta }_K,\varvec{\Sigma }_{spp}][\varvec{\Sigma }_{spp}]. \end{aligned} \end{aligned}$$

See Appendix A for the full statements of both the joint probit and Royle-Nichols occupancy models.

## Community confounding

### Restricted regression approach

We fit a restricted version of the each JSDM that orthogonalizes the fixed and random species effects. In the Royle-Nichols model, we express the species latent intensity and occupancy process conditionally as

13 $$\begin{aligned} \text {log}(\varvec{\lambda })&\sim \mathcal {N}(\varvec{X}\varvec{\delta } + (\varvec{I}-\varvec{P}_{\varvec{X}})\varvec{\eta }, \tau ^2\varvec{I})\text {, } \end{aligned}$$

14 $$\begin{aligned} \varvec{\eta }&\sim \mathcal {N}(\varvec{0}, \varvec{\Sigma }_{spp}\otimes \varvec{I}_n)\text {,} \end{aligned}$$

where $\varvec{P}_{\varvec{X}}$ is the projection matrix onto the column space of $\varvec{X}$. Likewise, in the probit model we specify $\Phi (\varvec{\psi })=\varvec{X}\varvec{\delta }+(\varvec{I}-\varvec{P}_{\varvec{X}})\varvec{\eta }$ and retain the same prior for $\varvec{\eta }$ as in (14). This specification forces the random species effects to explain patterns in the community that are orthogonal to the fixed effects. The latent variables and fixed effects in the LVM can also be orthogonalized. Writing (11) in matrix form, we have

15 $$\begin{aligned} \Phi (\varvec{\psi }_{k})=\varvec{X}'\varvec{\delta }_{k}+\varvec{L}\varvec{\theta }_k, \end{aligned}$$

where $\varvec{X}$ and $\varvec{L}$ are the matrices of covariates and latent variables vertically stacked across sites, respectively. If we assume common covariates across all *K* species, we can specify a restricted LVM as follows:

16 $$\begin{aligned} \Phi (\varvec{\psi }_{k})=\varvec{X}'\varvec{\delta }_{k}+(\varvec{I}-\varvec{P_X})\varvec{L}\varvec{\theta }_k. \end{aligned}$$

However, if covariates differ by species, i.e., $\varvec{X}=\varvec{X}_k$, then the posterior distribution of latent variables will differ by species. To retain a common posterior distribution of latent variables across all species, the latent variables need to be orthogonalized against all covariates among the *k* species,

17 $$\begin{aligned} \varvec{L}^{R}=\prod _{k=1}^{K}(\varvec{I}-\varvec{P}_{\varvec{X}_k})\varvec{L}. \end{aligned}$$

The specification of (17) is more restrictive than the orthogalization in the Royle-Nichols and probit model, and so we omit the LVM from our case study.

Hanks et al.^31^ showed that the restricted (13) and unrestricted (9) generalized linear mixed models GLMM are reparameterizations of the same model and derived the following relationship between the unconditional $\varvec{\delta }$ and conditional $\varvec{\beta }$ fixed effects:

18 $$\begin{aligned} \varvec{\delta }\equiv \varvec{\beta }+(\varvec{X}'\varvec{X})^{-1}\varvec{X}'\varvec{\eta }. \end{aligned}$$

Using (18), one can easily sample both sets of fixed effects by fitting either the restricted or unrestricted parameterization. We can also sample the covariance structure of the restricted random species effect from either model fit by drawing samples from the distribution

19 $$\begin{aligned} \varvec{\Sigma }_{spp,R}^{-1} \sim \text {Wishart}(\varvec{S}\nu +\varvec{\eta }'(\varvec{I}-\varvec{P_X})\varvec{\eta },\nu +n). \end{aligned}$$

Hence, regardless of which model is fit, we can obtain both the unconditional and conditional habitat effects as well as the unrestricted and restricted species covariance matrices.

### Measuring confounding

Hefley et al.^27^ showed how to assess confounding in SGLMM models by computing the Pearson correlation coefficient between each pair of covariates and eigenvectors from the spectral decomposition of the spatial covariance matrix. Likewise, Prates et al.^35^ proposed a test for spatial confounding that can be calculated prior to model fitting. We propose another approach relevant to our method that aids in interpretation. We compute the coefficient of determination of each covariate for species *k* regressed on the estimated random species effects. Because the latent intensities are unknown, the coefficents of determination of all covariates are derived quantities and can be computed at each iteration of the MCMC algorithm:

20 $$\begin{aligned} {R^2}^{(l)}(\varvec{x}_k) = \frac{SSR^{(l)}(\varvec{x}_k)}{SST(\varvec{x}_k)} = \frac{\left( \varvec{\Delta }^{(l)}\hat{\varvec{\theta }}^{(l)}-\bar{\varvec{x}}_k\right) '\left( \varvec{\Delta }^{(l)}\hat{\varvec{\theta }}^{(l)}-\bar{\varvec{x}}_k\right) }{\left( \varvec{x}_k-\bar{\varvec{x}}_k\right) '\left( \varvec{x}_k-\bar{\varvec{x}}_k\right) }, \end{aligned}$$

where $\bar{\varvec{x}}_k=\left( \bar{x}_k, \dots , \bar{x}_k\right) '$ is the mean of the covariate $\varvec{x}_k$ for species *k* repeated *n* times, $\varvec{\Delta }^{(l)}= \left( \varvec{\eta }_1^{(l)}, \dots , \varvec{\eta }_{K}^{(l)}\right)$ is a matrix of the random species effects sampled for MCMC iteration *l*, and $\hat{\varvec{\theta }}^{(l)}$ are estimated regression coefficients relating the estimated species intensities at iteration *l* to $\varvec{x}_k$. The posterior mean $\text {E}\left( R^2(\varvec{x}_k)|\varvec{Y}\right)$ provides a measure of community confounding for the covariate $\varvec{x}_k$ and can help identify which fixed effects will vary between the unrestricted and restricted models. Furthermore, we can use the global F-test of the linear relationship between $\varvec{x}_k$ and $\varvec{\Delta }$ to determine if confounding exists.

## Simulation study

We performed a simulation study to investigate the effects of community confounding and orthogonalization of the fixed and random species effects on model fitting. Specifically, we compared the effective sample sizes of $\varvec{\beta }$ and $\varvec{\eta }$ for three different models for confounded and unconfounded data with unrestricted and restricted parameterizations. The effective sample size (ESS) is the number of independent MCMC samples of a quantity and is a metric for measuring the sampling efficiency of an MCMC algorithm. Higher ESS are preferable as posterior distributions of quantities of interest can be obtained in fewer iterations.

We considered three models: The joint probit occupancy model, joint Royle-Nichols model, and joint normal model, which is derived from the scenario where $\varvec{\lambda }$ in the Royle-Nichols is known (e.g., species density data). For each model, 150 datasets were generated with the fixed and random species effects independent and another 150 datasets were generated with confounding between the fixed and random species effects. To induce confounding between the fixed and random species effect, we expressed one covariate of the first species as a linear combination of the random species effects (i.e., $\varvec{x}_1=\varvec{\Delta }\varvec{\theta }$).

Because the ratio of the random effects and random error magnitude is known to affect the severity of confounding in the spatial context^29,31,35^, we varied the magnitude of the random species effect in each model while holding the random error magnitude constant. Specifically, each dataset was subdivided into thirds with 50 datasets simulated to have small, medium, and large random species effects relative to the random error.

All 900 simulated datasets across models and confounding levels were for $K=2$ species across $n=50$ sites with $J=10$ occasions per site for the occupancy models. The correlation between the two species was allowed to vary for each dataset. Each habitat design matrix included an intercept and one continuous covariate. Each MCMC algorithm was run for a burn-in period of $L=10000$ to ensure convergence. The next $L=10000$ iterations were used to calculate the posterior quantities in Table 1. Code for performing the simulation study in R are available in the supplementary electronic files.

**Table 1:** Summary of simulations results.

Model	Data	$\varvec{\beta }$ ESS Ratio	$\varvec{\beta }$ Mean ESS	$\varvec{\eta }$ ESS Ratio	$\varvec{\eta }$ Mean ESS	$\text {E}\left( R^2(\varvec{x}_1)|\varvec{Y}\right)$	Rejection rate
Normal	Unconfounded	18.69	5143	6.20	5670	0.04	0.01
Normal	Confounded	8.67	4219	5.79	4800	0.51	0.87
Probit	Unconfounded	1.73	959	1.08	534	0.04	0.00
Probit	Confounded	1.96	444	1.23	293	0.19	0.63
Royle-Nichols	Unconfounded	0.81	232	0.98	307	0.04	0.00
Royle-Nichols	Confounded	0.80	186	0.98	301	0.18	0.51

All results are averaged across 3 magnitudes of random species effects and 50 simulated datasets. ESS Ratio is the effective sample size of the restricted parameterizations over the unrestricted and the mean ESS is the average of the two. $\text {E}\left( R^2(\varvec{x}_1)|\varvec{Y}\right)$ is the posterior mean $R^2$ of confounding for species 1 continuous habitat covariate. Rejection rate is the portion of times the the posterior mean p-value from overall F-test of a linear relationship between $\varvec{x}_1$ and $\varvec{\Delta }$ was below 0.05.

For both $\varvec{\beta }$ and $\varvec{\eta }$, ESS was lower on average for the confounded data than the unconfounded data for all three models demonstrating the negative impacts confounding can have on model fitting. For all three models, the computational impact of fitting the restricted parameterization did not differ depending on whether confounding exists or not. In the case of the normal and probit models, fitting the restricted parameterization improved ESS for both $\varvec{\beta }$ and $\varvec{\eta }$, although the gains were much greater for the normal model. On the other hand, the restricted parameterization of the Royle-Nichols model did not improve ESS for $\varvec{\beta }$ or $\varvec{\eta }$. The success of our method for detecting community confounding differed across models. The method was most powerful for the normal model followed by the probit and Royle-Nichols models.

## Camera trap survey

### Study area

**Figure 1:**
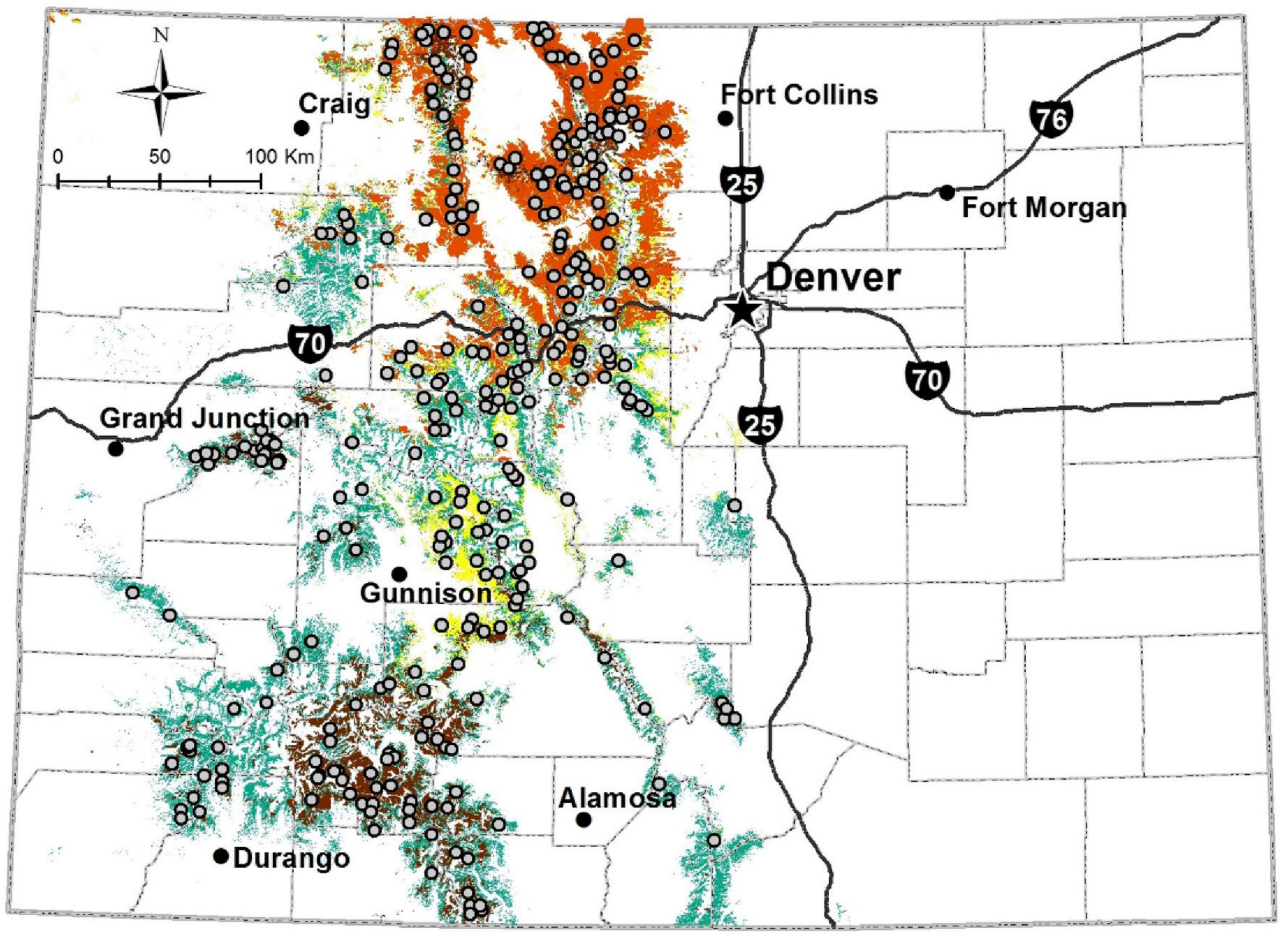
Randomly selected sampling sites (gray circles) where passive infrared game cameras were deployed in spruce-fir (green) and lodgepole pine (yellow) forests in Colorado, USA, 2013-2014. Brown and orange are the approximate extents of spruce beetle and mountain pine beetle impacts in spruce-fir and lodgepole pine forests, respectively, as of 2014. Reprinted from “Mammalian responses to changed forest conditions resulting from bark beetle outbreaks in the southern Rocky Mountains,” by J. S. Ivan, 2018, Ecosphere, 9(8), e02369. Figure produced with ArcMap 10.7 available at: https://desktop.arcgis.com/en/arcmap/10.7/get-started/setup/arcgis-desktop-quick-start-guide.html.

We analyzed data arising from a study area comprised of subalpine forests in the state of Colorado between 2590 and 3660 m elevation (Fig. 1). Sites were restricted to public lands managed by the United States Forest Service, National Park Service, Bureau of Land Management, and Colorado State Forest Service. Forests in our study area were primarily composed of Lodgepole pine (*Pinus contorta*), Engelmann spruce (*Picea engelmannii*), and subalpine fir (*Abies lasiocarpa*). Lodgepole pine was dominant at lower elevations as well as higher elevations that were drier and/or on south-facing slopes; high elevation regions that had cool north-facing slopes were co-dominated by Engelmann spruce and subalpine fir. Lodgepole pine is restricted to the northern two-thirds of Colorado, so all sites in the southern region of the study area were Engelmann spruce, subalpine fir co-dominated. Quaking aspen (*Populus tremuloides*), Douglas-fir (*Pseudotsuga menziesii*), bristlecone pine (*Pinus aristata*), limber pine (*Pinus flexilis*), and blue spruce (*Picea pungens*) were also present at some sites. Mean July and January temperature across the study area were 14 and − 6.1 °C respectively. All camera data were collected during summers 2013–2014.

### Sampling design

The primary goal of Ivan et al.^50^ was to assess mammalian responses to bark beetle outbreaks, thus sites were randomly selected to facilitate inference on the beetle outbreak covariates. Beetle outbreak covariates included the number of years since the initial outbreak (YSO) and the severity of the outbreak measured by mean overstory mortality (severity). The sample of $n=300$, 1 km^2^ sites was evenly split across the two dominant forest types, spruce-fir and lodgepole pine. Additional environmental covariates were collected at each site, and a description of these is included in Appendix B.

Passive infrared camera traps (Reconyx PC800, Holmen, Wisconsin, USA) were deployed near the center of each site. Cameras were approximately 0.5 m above the ground and pointed toward a lure tree 4–5 m away^52^. The setup was designed to maximize detections of both large and small-bodied mammals in the local community while minimizing attraction of individuals from outside the sampling region of the site. The sampling regions were likely not closed to immigration/emigration; thus, we interpret elevated detections at a site as more individuals using, as opposed to occupying, that site^53^. For additional details regarding the sampling design and study area see Ivan et al.^50^.

### Model fitting

We fit both the Royle-Nichols and probit occupancy models to the camera trap data binned into 20 two-day occasions because simulations showed this was the number of replications needed to identify a quadratic effect of occasion on individual detection probability. Not all cameras were operational for the entire 40 day sampling period, and thus the number of occasions varied from 7-20. We discarded four sites at which the camera was operational for less than one occasion. We also discarded another 12 sites that had been infested by bark beetles for more than 10 years. Ivan et al.^50^ truncated the bark beetle infestation covariate at 10 years because estimates of response curves beyond 10 years would be unreliable with so few sites. The final sample size was $n=284$ sites. We built distribution models for the 13 species for which Ivan et al.^50^ performed a single species analysis; several rare species were excluded from analysis due to insufficient detections. We note, however, that these rare species parameters may be identifiable in the joint model as has been the case in previous studies^2,47,54–57^. Our final dataset then included 3692 unique encounter histories at $n=284$ sites, stacked across $K=13$ species.

Ivan et al.^50^ used a sequential procedure similar to that described in Lebreton et al.^58^ to select the covariates in the occupancy and detection processes for each species. We adopted their detection model and used the same covariates but a different set of basis functions for YSO. Ivan et al.^50^ treated YSO as a grouping variable and considered probability of use response curves that allowed for cubic associations and delayed responses to bark beetle infestation. Multiple response curves were model averaged to produce predictive YSO response curves for each species. We used orthogonal polynomial basis functions for the YSO variable in the species intensity models. The basis functions included a linear (YSO1) and quadratic (YSO2) effect. Appendix E provides a full description of the intensity and detection models. All continuous covariates were scaled to have mean 0 and variance 1.

We fit all models using Markov chain Monte Carlo (MCMC). To improve mixing and predictive ability, we regularized the coefficients $\varvec{\beta }$ and $\varvec{\alpha }$ with informative priors: $\varvec{\beta } \sim \mathcal {N}(\varvec{0}, \varvec{I})$ and $\varvec{\alpha } \sim \mathcal {N}(\varvec{0}, \varvec{I})$^26^. We specified a vague prior of $\varvec{\Sigma }^{-1}_{spp} \sim \text {Wishart}(15, (15\varvec{I})^{-1})$ for the species variance-covariance matrix^59^. For the Royle-Nichols model, we used Gibbs sampling based on conjugate priors for parameters $\varvec{\Sigma }_{spp}$, $\varvec{\eta }$, and $\varvec{\beta }$ and Metropolis-Hastings updates for $\varvec{N}$, $\varvec{\lambda }$, and $\varvec{\alpha }$. Derivations of the conjugate full-conditional distributions are provided in Appendix C with details about the Metropolis-Hastings updates. We tuned the Metropolis-Hastings updates so that acceptance rates varied between 20 and 40% for $\varvec{\alpha }$, $\varvec{N}$, and $\varvec{\lambda }$. Using data augmentation^17,43–45^, all the parameters of the probit model can be sampled with Gibbs updates.

We set $\tau ^2 = 2.25$ in both (9) and (13). This choice was supported by the asymptotic equivalence between Poisson and logistic regression. In a generalized occupancy model, the latent probability of occupancy is specified as $\text {logit}(\psi _i) \sim \mathcal {N}(\varvec{x}'_i\varvec{\beta }, \tau ^2)$. Hanson et al.^60^ investigated the relationship between the prior on $\varvec{\beta }$ and induced prior on the latent probability of success $\psi _i$ in logistic regression; their work showed that specifying an uninformative normal prior on $\varvec{\beta }$ (i.e., setting $\tau ^2$ large) induces a U-shaped prior for $\psi _i$ with most of the density concentrated near 0 and 1. Broms et al.^14^ recommended setting $\tau ^2 = 2.25$ in occupancy models, which results in a relatively flat prior for $\psi$. For rare species, $\lambda _i$ in (9) and (13) is analogous to $\psi _i$, and specifying a variance of $\tau ^2 = 2.25$ is minimally informative.

Baddeley^61^ motivated the asymptotic equivalence of Poisson and logistic regression in a spatial context where counts of points from a non-homogeneous Poisson process are recorded in a lattice; they showed that, as the grid cells of the lattice become infinitesimally small, the inference yielded from Poisson and logistic regression are equivalent. This result can be applied more generally to any dataset where there is a high proportion of zero counts. We demonstrate the asymptotic equivalence between Poisson and logistic regression in the Royle-Nichols model in Appendix D.

We ran the MCMC algorithm for $L=50000$ iterations, and discarded the first 12500 iterations as burn-in. We fit an SDM, unrestricted JSDM, and restricted JSDM of both the Royle-Nichols and probit occupancy models. The “Results” section presents inference for regression coefficients for all six model fits.

### Results

**Figure 2:**
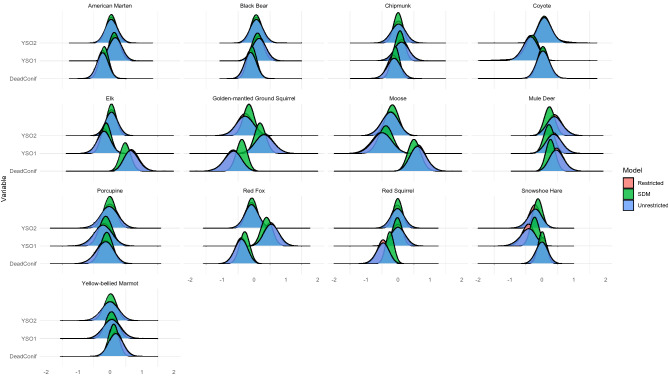
Marginal posterior distributions of infestation regression parameters. Posterior distributions shown are from the probit SDM, unrestricted JSDM, and restricted JSDM. DeadConif is the overstory mortality percentage, a proxy for severity of bark beetle infestation. YSO1 is the linear effect of the number of years since a site was infested with bark beetles. YSO2 is the quadratic effect. Figure created in R 4.1.2^62^.

Ivan et al.^50^ fit SDMs to infer changes in mammalian use of stands impacted by the bark beetle epidemic. The impact of bark beetle damage was measured by years since initial infestation (YSO) and severity of outbreak quantified by mean overstory mortality (DeafConif). The posterior distributions of the regression coefficients varied between the probit SDM and unrestricted JSDM, although the magnitude of difference differed by species (Fig. 2). The posterior variances of the SDM regression coefficients were smaller than the unrestricted JSDM. Posterior variances and means of the restricted probit JSDM regression coefficients quite similar to those from unrestricted JSDM. The only noticeable difference between the unrestricted and restricted regression coefficients was that the restricted coefficients had slightly smaller posterior variances on average.

**Figure 3:**
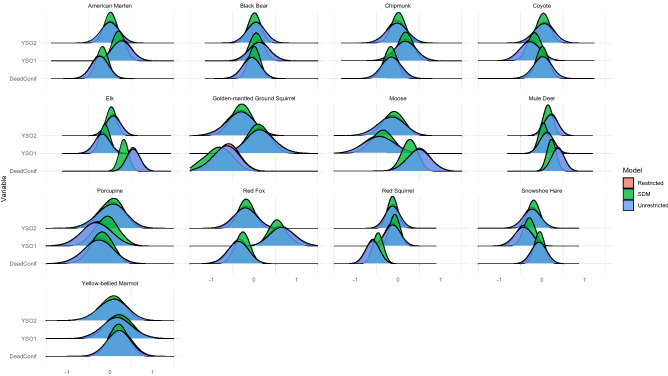
Marginal posterior distributions of infestation regression parameters. Posterior distributions shown are from the Royle-Nichols SDM, unrestricted JSDM, and restricted JSDM. DeadConif is the overstory mortality percentage, a proxy for severity of bark beetle infestation. YSO1 is the linear effect of the number of years since a site was infested with bark beetles. YSO2 is the quadratic effect. Figure created in R 4.1.2^62^.

As with the probit modeling results, posterior distributions of the regression coefficients in the Royle-Nichols SDM were more concentrated near zero than those of the JSDM (Fig. 3). Also, posterior distributions of the restricted JSDM regression coefficients were slightly tighter and centered closer to zero.

**Figure 4:**
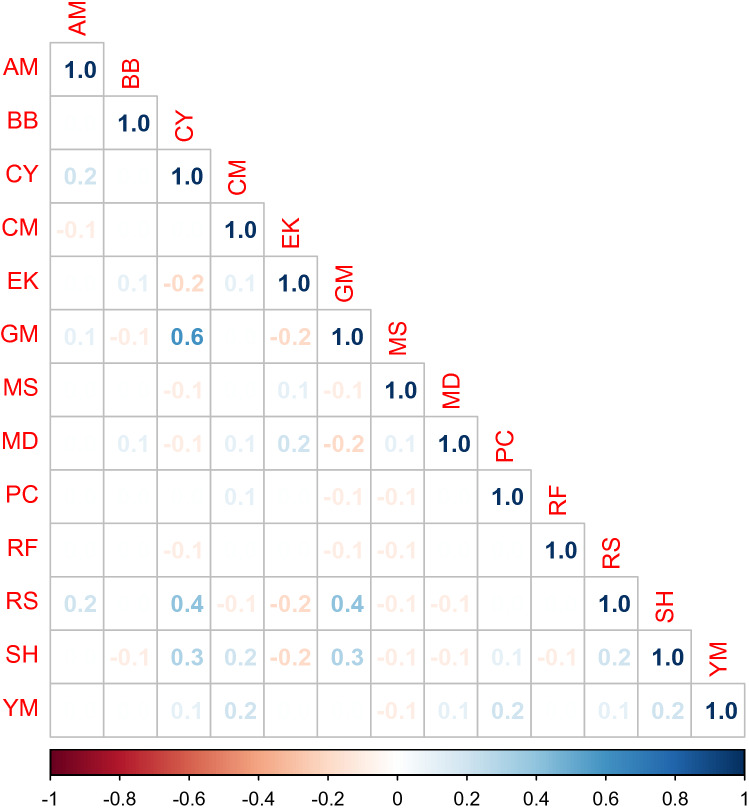
Posterior mean of species correlation matrix. Estimates are from the Royle-Nichols unrestricted joint species distribution model. AM = American Marten, BB = Black Bear, CY = Coyote, CM = Chipmunk spp., Ek = Elk, GM = Golden-mantled Ground Squirrel, MS = Moose, MD = Mule Deer, PC = Porcupine, RF = Red Fox, RS = Red Squirrel, SH = Snowshoe Hare, YM = Yellow-bellied Marmot. Figure created in R 4.1.2^62^.

We calculated the unrestricted and restricted posterior correlation matrices for both the probit and Royle-Nichols models. Pairwise differences between each entry of the posterior mean of the four correlation matrices were bounded between $(-0.2, 0.2)$, so only the correlation matrix of the unrestricted Royle-Nichols model is shown (Fig. 4). The posterior distributions of the pairwise correlations all overlapped zero except for the pairwise correlations between coyotes and golden-mantled ground squirrels, coyotes and red squirrels, and golden-mantled ground squirrels and red squirrels. In the restricted probit JSDM, the correlations between coyotes and snowshoe hares, and snowshoe hares and red squirrels also did not overlap zero.

We calculated the posterior $R^2$ of confounding for each covariate in each species specific model as described in (20). All posterior $R^2$ were below 0.05 for both the Royle-Nichols and probit models giving no indication of community confounding for all covariates considered.

## Discussion

We found that confounding between the fixed and random species effects can reduce sampling efficiency in MCMC algorithms and that orthogonalizing the fixed and random species effects can alleviate this problem when fitting some joint species distribution models. In the simulation study, we discovered that, even when the data were not confounded, orthogonalizing the fixed and random species effects still conferred a computational benefit for the normal and probit model. This was also true for our case study where the mean effective sample size of the conditional habitat effects $\varvec{\beta }$ in the probit model was 32% larger when fit with the restricted parameterization. The effective sample size of $\varvec{\eta }$ in the probit model was 3% greater for the restricted parameterization.

The case study indicated that inference on species-environment associations in occupancy models can change based on whether the distribution model accounts for community structure. Orthogonalizing the fixed and random species effects in the probit and Royle-Nichols model slightly reduced but did not nullify the differences as in the case for normal data. The similarity between the restricted and unrestricted JSDM coupled with the lack of evidence for community confounding suggests additional mechanisms lead inference in SDMs and JSDMs to differ, a finding consistent with Caradima et al.^63^. Overall, there was still large agreement in posterior inference produced by the SDM and JSDMs for both occupancy models. In additional simulation studies on the probit and Rolye-Nichols occopancy models, we found that community confounding can lead to larger differences between the SDM and unrestricted JSDM and that the restricted JSDM again mitigates but rarely nullifies these differences.

We were also interested in whether the Royle-Nichols model could identify additional associations compared with the probit model. The Royle-Nichols model measures associations conditional on an intensity process rather than an occupancy process, and intensity is likely a function of additional factors beyond those influencing occupancy^19–21^. For the camera trap data, the opposite was true, in that the probit model identified more environmental-species and species-species associations. One possible explanation for this is that the probit model is more parsimonious which sharpens posterior distributions.

A related method to restricted regression, which orthogonalizes the fixed and random effects, is principal components regression, which performs an orthogonalization procedure solely among the fixed effects. To motivate their similarities, consider a simpler case where the latent intensities, $\varvec{\lambda }$, of the *K* species in our community were known. We could construct *K* regression models for predicting each species intensity as follows:

21 $$\begin{aligned} \varvec{\lambda }_k = \varvec{X}_k\varvec{\beta }_k + \varvec{\Lambda }_{-k}\varvec{\eta }_k + \varvec{\varepsilon }, \end{aligned}$$

where $\varvec{\Lambda }_{-k} = \left( \varvec{\lambda }_1, \dots , \varvec{\lambda }_{k-1}, \varvec{\lambda }_{k+1}, \dots , \varvec{\lambda }_{K} \right)$ is a matrix of the $K-1$ other species intensities. If $\varvec{X}_k$ and $\varvec{\Lambda }_{-k}$ were highly collinear, principal component regression might be applied. Principal components regression is so named because it decomposes the variation explained by $\varvec{X}_k$ and $\varvec{\Lambda }_{-k}$ into $p = p_1 + p_2$ principal components, $\varvec{\Gamma }_k = \left( \varvec{\gamma }_1, \dots , \varvec{\gamma }_p \right),$ where $p_1$ and $p_2$ are the number of columns of $\varvec{X}_k$ and $\varvec{\Lambda }_{-k}$ respectively. The *p* principal components retain all the information explained by $\varvec{X}_k$ and $\varvec{\Lambda }_{-k}$ but are orthogonal. The regression model

22 $$\begin{aligned} \varvec{\lambda }_k&= \varvec{W}_k\varvec{\theta } + \varvec{\varepsilon }, \end{aligned}$$

23 $$\begin{aligned} \varvec{W}_k&= \left( \varvec{X}_k, \varvec{\Lambda }_{-k} \right) \varvec{\Gamma }_k, \end{aligned}$$

often improves sampling efficiency and can recover the posterior means and variances of $\varvec{\beta }_k$ and $\varvec{\eta }_k$ in (21). However, inference on $\varvec{\beta }_k$ and $\varvec{\eta }_k$ is often adjusted by truncating off the last $p-r$, for $r<p$, eigenvectors of $\varvec{\Gamma }_k$ and employing the new design matrix

24 $$\begin{aligned} \varvec{W}^{\star }_k&= \left( \varvec{X}_k, \varvec{\Lambda }_{-k} \right) \varvec{\Gamma }^{\star }_k, \end{aligned}$$

25 $$\begin{aligned} \varvec{\Gamma }^{\star }_k&= \left( \varvec{\gamma }_1, \dots , \varvec{\gamma }_r \right) . \end{aligned}$$

By retaining only the first *r* principal components, the smallest sources of variation are ignored in the estimation of $\varvec{\beta }_k$ and $\varvec{\eta }_k$. Jeffers^64^ implemented this approach truncating off the last 7 of 13 principal components to adjust the estimates of regression coefficients relating various tree characteristics to maximum compressive strength. Other studies have selected a subset of principal components based on their strength of association with the response variable^65–68^. In some cases, the coefficient estimates from these reduced rank approaches appeared more tenable than those from the full rank specifications based on known physical relationships between the predictors and response. Thus, like restricted regression, principal components regression can be used for solely computation purposes or to adjust inference.

Recently, concerns regarding the coverage properties of the fixed effects estimator under restricted regression have been expressed^36,69^. For example, Zimmerman and Ver Hoef^69^ showed that applying any restricted regression method to a SGLMM leads to frequentest coverage of the fixed effects that is lower than the corresponding non-spatial model. Similarly, Khan and Calder^36^ found that when fitting a restricted version of the SGLMM with an intrinsic conditional autoregressive prior, credible intervals of the fixed effects from the restricted model were generally nested inside those yielded by the non-spatial model. Given these results, both Zimmerman and Ver Hoef^69^ and Khan and Calder^36^ recommended reverting to inference from the non-spatial model, rather than that of the restricted SGLMM, when inference from the unrestricted SGLMM appears untenable.

We did not observe the same pattern in our restricted JSDM but found the length of credible intervals of the restricted regression coefficients to generally be between that of the SDM and unrestricted JSDM. Nonetheless, if higher coverage is desired, one can always extract the conditional coefficients from the restricted JSDM while still benefiting from the increased stability that results from orthogonalizing the fixed and random effets. When deciding between inference from the restricted and unrestricted JSDM, one should also consider the random species effects $\varvec{\eta }$. Because the random effect $\varvec{\eta }$ is rarely of interest in spatial applications, there has been little investigation on the inferential impacts of restricted regression on $\varvec{\eta }$. Such investigation, however, may be helpful in determining the appropriateness of restricted regression for JSDMs.

There are several conceptual facets to consider regarding the application of restricted regression in joint species distribution modeling. Frequently, JSDMs are described as accounting for residual correlations between species that cannot be explained by the environmental covariates^4,5,63^. We have shown, however, that in some JSDMs, the random species effect can explain variation that is collinear with environmental covariates. Only in the restricted JSDM, does the random species effect explain variation that is residual to the environmental covariates attributing all contested sources of variation to the fixed effect. Yet, given that species environmental requirements can fluctuate based on their symbiotic relationships, one might argue that interplay between the environmental effects and interspecies dependence is ecology warranted. Therefore, any method that removes the conditional nature of these effects like restricted regression is inappropriate.

JSDMs have been described as correcting our knowledge of species-environment relationship by accounting for interspecies dependence^5^. Poggiato et al.^5^ argued that JSDMs help us better quantify uncertainty regarding species-environment relationships, but they cannot explain discrepancies in a species theoretical and realized niche. We agree that phenomenological JSDMs should not be used to disentangle the marginal effects of environment and interspecies dependence on species distributions and would recommend the development of mechanistic models to investigate interspecies-environment associations.

Experimental methods and modeling techniques for alleviating confounding have been proposed in ecology. Hefley et al.^70^ showed that replicate populations can help disentangle confounded fixed and random effects. In the context of joint species distribution modeling, replication involves analyzing several communities simultaneously, which is often infeasible. Hefley et al.^70^ also recommended explicit population models rather than phenomenological regression-based models for analysis of temporally confounded count data. Similarly, Fieberg et al.^71^ advocated for mechanistic models guided by causal diagrams for analyzing temporally confounded animal movement data. An avenue of future research for joint species distribution modeling is to compare inference from phenomenological regression-based models, such as the one proposed here, with that of models that explicitly include ecological mechanisms such as competitive exclusion, mutualism, and predation. Because community and temporal confounding have the same mathematical framework, mechanistic models are a promising solution for confounded multispecies data.

In summary, we specified a JSDM that accounts for interspecies dependence at the intensity level, and examined how inference from the joint model differed from the joint probit model. We performed a simulation study on three JSDMs to examine the computational difficulties associated with community confounding and investigated whether orthogonalizing the fixed and random species effect could alleviate these difficulties. Further, we considered how inference in both occupancy models differed depending on the assumed community structure. Lastly, we discussed how joint species distribution modeling is distinct from spatial and time series applications in that the random effect is almost always of inferential interest, and hence, adjustments to the regression coefficients, $\varvec{\beta }$, and random effects, $\varvec{\eta }$, should both be considered. Our main conclusion is that, even for researchers who desire inference solely on the conditional relationship between the fixed species-environment and random species effects, fitting the JSDM with a restricted parameterization can give computational benefits.

## Supplementary Information

Supplementary Information.

Supplementary Dataset 1.

## Data Availability

The data are available in the Supplementary Information files.

## Code availability

All algorithms and code for fitting and analyzing results from the six model variants are available in the Supplementary Information files. All MCMC algorithms and analyses were coded in R 4.1.2^62^.
