# Supplementary material for: Community confounding in joint species distribution models
Source: Sci Rep. 2022 Jul 18;12:12235. doi: 10.1038/s41598-022-15694-6 (PMC9294001; doi:10.1038/s41598-022-15694-6)
Supplement: Supplementary file 1 — Supplementary Information. [file 41598_2022_15694_MOESM1_ESM.pdf]

Appendix: Community Confounding in Joint Species Distribution Models

A JOINT OCCUPANCY MODELS

A.1 ROYLE-NICHOLS

$$
\begin{aligned}
&y_{ijk} \sim \text{Bernoulli}(\rho_{ijk}),\ \rho_{ijk} = 1 - (1 - r_{jk})^{N_{ik}},\ \text{logit}(r_{jk}) = g(j, \boldsymbol{\alpha}_k), && \text{Response}\\
&N_{ik} \sim \text{Pois}(\lambda_{ik}), && \text{Process}\\
&\log(\boldsymbol{\lambda}) = \mathcal{N}(\boldsymbol{X\beta} + \boldsymbol{\eta}, \tau^2 \boldsymbol{I}) && \text{Process}\\
&\boldsymbol{\eta} \sim \mathcal{N}(\boldsymbol{0}, \boldsymbol{\Sigma}_{spp} \otimes \boldsymbol{I}_n), && \text{Process}\\
&\boldsymbol{\alpha} \sim \mathcal{N}(\boldsymbol{\mu_\alpha}, \boldsymbol{\Sigma_\alpha}), && \text{Prior}\\
&\boldsymbol{\beta} \sim \mathcal{N}(\boldsymbol{\mu_\beta}, \boldsymbol{\Sigma_\beta}), && \text{Prior}\\
&\boldsymbol{\Sigma}_{spp}^{-1} \sim \text{Wishart}(\boldsymbol{S}/\nu, \nu). && \text{Prior}
\end{aligned}
$$

A.2 PROBIT

$$
\begin{aligned}
&y_{ijk} \sim \begin{cases} \text{Bernoulli}(p_{ijk}), & z_{ik} = 1 \\ 0, & z_{ik} = 0 \end{cases}, && \text{Response}\\
&\Phi(\boldsymbol{p}) = \boldsymbol{W\alpha}, && \text{Process}\\
&z_{ik} \sim \text{Bernoulli}(\psi_{ik}), && \text{Process}\\
&\Phi(\boldsymbol{\psi}) = \mathcal{N}(\boldsymbol{X\beta} + \boldsymbol{\eta}, \boldsymbol{I}) && \text{Process}\\
&\boldsymbol{\eta} \sim \mathcal{N}(\boldsymbol{0}, \boldsymbol{\Sigma}_{spp} \otimes \boldsymbol{I}_n), && \text{Process}\\
&\boldsymbol{\alpha} \sim \mathcal{N}(\boldsymbol{\mu_\alpha}, \boldsymbol{\Sigma_\alpha}), && \text{Prior}\\
&\boldsymbol{\beta} \sim \mathcal{N}(\boldsymbol{\mu_\beta}, \boldsymbol{\Sigma_\beta}), && \text{Prior}\\
&\boldsymbol{\Sigma}_{spp}^{-1} \sim \text{Wishart}(\boldsymbol{S}/\nu, \nu). && \text{Prior}
\end{aligned}
$$

B MCMC IMPLEMENTATION

For the sites at which we never detected species k, we sample N_{ik} using a Poisson Gibbs update.

$$
\begin{aligned}
&[N_{ik}|\cdot\,] \propto \left(\prod_{j=1}^{J_i}[y_{ijk}|N_{ik}, \boldsymbol{\alpha}_k]\right)[N_i|\lambda_{ik}],\\
&\propto \left(\prod_{j=1}^{J_i}\{1-(1-r_{ij})^{N_{ik}}\}^{y_{ijk}}\{(1-r_{ij})^{N_{ik}}\}^{1-y_{ijk}}\right)\frac{\left(\lambda_{ik}\right)^{N_{ik}}}{N_{ik}!},\\
&\text{For } \boldsymbol{y}_{ik}=\mathbf{0}, \text{ we have,}\\
&\propto \left(\prod_{j=1}^{J_i}(1-r_{ij})\right)^{N_{ik}}\frac{\left(\lambda_{ik}\right)^{N_{ik}}}{N_{ik}!},\\
&\propto \frac{\left(\prod_{j=1}^{J_i}(1-r_{ij})\lambda_{ik})\right)^{N_i}}{N_{ik}!},\\
&\propto \frac{\exp\left(\prod_{j=1}^{J_i}(1-r_{ij})\lambda_{ik}\right)\left(\prod_{j=1}^{J_i}(1-r_{ij})\lambda_{ik})\right)^{N_i}}{N_{ik}!},\\
&= \text{Pois}\left(\prod_{j=1}^{J_i}(1-r_{ij})\lambda_{ik}\right).
\end{aligned}
$$

For any other sequence of detections, the full-conditional distribution for N_{ik} is not tractable, and we use Metropolis-Hastings based on the following MH ratio:

$$
\text{MH} = \frac{\left(\prod_{j=1}^{J_i}[y_{ijk}|N_{ik}^{(\star)}, \boldsymbol{\alpha}_k^{(l-1)}]\right)[N_{ik}^{(\star)}|\lambda_{ik}^{(l-1)}][N_{ik}^{(\star)}|N_{ik}^{(l-1)}]}{\left(\prod_{j=1}^{J_i}[y_{ijk}|N_{ik}^{(l-1)}, \boldsymbol{\alpha}_k^{(l-1)}]\right)[N_{ik}^{((l-1)}|\lambda_{ik}^{(l-1)}][N_{ik}^{(l-1)}|N_{ik}^{(\star)}]},
$$

where the distribution $[N_{ik}^{(\star)}|N_{ik}^{(l-1)}]$ is the proposal; we used a zero truncated Poisson to ensure that $N_{ik} > 0$ at sites for which there was at least one detection.

The full-conditional distribution for λ is also irregular, and we update the λ_{ik} using Metropolis-Hastings with a normal random walk proposal. The MH ratio is as follows:

$$
\text{MH} = \frac{[N_{ik}^{(l-1)}|\lambda_{ik}^{(\star)}][\lambda_{ik}^{(\star)}|\boldsymbol{\beta}_k^{(l-1)}, \eta_{ik}^{(l-1)}]}{[N_{ik}^{(l-1)}|\lambda_{ik}^{(l-1)}][\lambda_{ik}^{(l-1)}|\boldsymbol{\beta}_k^{(l-1)}, \eta_{ik}^{(l-1)}]},
$$

$$
\left[\log\lambda_{ik}|\boldsymbol{\beta}_k, \eta_{ik}^{(l-1)}\right] = \mathcal{N}(\boldsymbol{x}_{ik}'\boldsymbol{\beta}_k^{(l-1)} + \eta_{ik}^{(l-1)}, \tau^2).
$$

The priors for $\boldsymbol{\beta}$, $\boldsymbol{\eta}$, and $\boldsymbol{\Sigma}_{spp}$ are conjugate, and the full-conditional distributions are

tractable. Below are the derivations.

$$
\begin{aligned}
&[\boldsymbol{\beta}|\cdot] \propto [\boldsymbol{\lambda}|\boldsymbol{\beta}, \boldsymbol{\eta}, \boldsymbol{\Sigma}_{spp}][\boldsymbol{\beta}], \\
&\propto \exp\left(-\frac{1}{2}(\log(\boldsymbol{\lambda}) - (\boldsymbol{X}\boldsymbol{\beta} + \boldsymbol{\eta}))'(\tau^2 \boldsymbol{I})^{-1}(\log(\boldsymbol{\lambda}) - (\boldsymbol{X}\boldsymbol{\beta} + \boldsymbol{\eta}))\right) \\
&\times \exp\left(-\frac{1}{2}(\boldsymbol{\beta} - \boldsymbol{\mu}_{\boldsymbol{\beta}})'\boldsymbol{\Sigma}_{\boldsymbol{\beta}}^{-1}(\boldsymbol{\beta} - \boldsymbol{\mu}_{\boldsymbol{\beta}})\right), \\
&\propto \exp\left(-\frac{1}{2}(-2\boldsymbol{\beta}'((\tau^2 \boldsymbol{I})^{-1}(\log(\boldsymbol{\lambda}) - \boldsymbol{\eta}) + \boldsymbol{\Sigma}_{\boldsymbol{\beta}}{}^{-1}\boldsymbol{\mu}_{\boldsymbol{\beta}}) + \boldsymbol{\beta}'(\boldsymbol{X}'(\tau^2 \boldsymbol{I})^{-1}\boldsymbol{X} + \boldsymbol{\Sigma}_{\boldsymbol{\beta}}{}^{-1})\boldsymbol{\beta})\right), \\
&\implies [\boldsymbol{\beta}|\cdot] = \mathcal{N}(\boldsymbol{A}^{-1}\boldsymbol{b}, \boldsymbol{A}^{-1}), \\
&\text{where,} \\
&\boldsymbol{A}^{-1} = \tau^{-2}\boldsymbol{X}'\boldsymbol{X} + \boldsymbol{\Sigma}_{\boldsymbol{\beta}}{}^{-1}, \\
&\boldsymbol{b} = \tau^{-2}\boldsymbol{X}'(\log(\boldsymbol{\lambda}) - \boldsymbol{\eta}) + \boldsymbol{\Sigma}_{\boldsymbol{\beta}}{}^{-1}\boldsymbol{\mu}_{\boldsymbol{\beta}}.
\end{aligned}
$$

$$
\begin{aligned}
&[\boldsymbol{\eta}|\cdot] \propto [\boldsymbol{\lambda}|\boldsymbol{\beta}, \boldsymbol{\eta}, \boldsymbol{\Sigma}_{spp}][\boldsymbol{\eta}], \\
&\propto \exp\left(-\frac{1}{2}(\log(\boldsymbol{\lambda}) - (\boldsymbol{X}\boldsymbol{\beta} + \boldsymbol{\eta}))'((\tau^2 \boldsymbol{I})^{-1}(\log(\boldsymbol{\lambda}) - (\boldsymbol{X}\boldsymbol{\beta} + \boldsymbol{\eta}))\right) \\
&\times \exp\left(-\frac{1}{2}(\boldsymbol{\eta}(\boldsymbol{\Sigma}_{spp} \otimes \boldsymbol{I}_n)^{-1}\boldsymbol{\eta})\right), \\
&\propto \exp\left(-\frac{1}{2}(-2\boldsymbol{\eta}'((\tau^2 \boldsymbol{I})^{-1}(\log(\boldsymbol{\lambda}) - \boldsymbol{X}\boldsymbol{\beta}) + \boldsymbol{\eta}'((\tau^2 \boldsymbol{I})^{-1} + (\boldsymbol{\Sigma}_{spp} \otimes \boldsymbol{I}_n)^{-1})\boldsymbol{\eta})\right), \\
&\implies [\boldsymbol{\eta}|\cdot] = \mathcal{N}(\boldsymbol{A}^{-1}\boldsymbol{b}, \boldsymbol{A}^{-1}), \\
&\text{where,} \\
&\boldsymbol{A}^{-1} = \tau^{-2}\boldsymbol{I} + (\boldsymbol{\Sigma}_{spp} \otimes \boldsymbol{I}_n)^{-1}, \\
&\boldsymbol{b} = \tau^{-2}(\log(\boldsymbol{\lambda}) - \boldsymbol{X}\boldsymbol{\beta}).
\end{aligned}
$$

Denote $\boldsymbol{\Delta} = \begin{pmatrix} \boldsymbol{\eta}_1 \ldots, \boldsymbol{\eta}_K \end{pmatrix}_{n \times K}$.

$[\boldsymbol{\Sigma}_{spp}|\cdot] \propto [\boldsymbol{\eta}|\boldsymbol{\Sigma}_{spp}][\boldsymbol{\Sigma}_{spp}]$,

$\propto |\boldsymbol{\Sigma}_{spp} \otimes \boldsymbol{I}_n|^{-\frac{1}{2}} \exp\left(-\frac{1}{2}(\boldsymbol{\eta}'(\boldsymbol{\Sigma}_{spp} \otimes \boldsymbol{I}_n)^{-1}\boldsymbol{\eta})\right)$

$\times |\boldsymbol{\Sigma}_{spp}|^{-(\nu+K+1)/2} \exp\left(-\frac{1}{2}\mathrm{tr}(\boldsymbol{S}\boldsymbol{\Sigma}_{spp}^{-1})\right)$,

$\propto |\boldsymbol{\Sigma}_{spp}|^{-\frac{n}{2}} |\boldsymbol{I}_n|^{-\frac{K}{2}} \exp\left(-\frac{1}{2}(\boldsymbol{\eta}'(\boldsymbol{\Sigma}_{spp}^{-1} \otimes \boldsymbol{I}_n)\boldsymbol{\eta})\right)$

$\times |\boldsymbol{\Sigma}_{spp}|^{-(\nu+K+1)/2} \exp\left(-\frac{1}{2}\mathrm{tr}(\boldsymbol{S}\boldsymbol{\Sigma}_{spp}^{-1})\right)$, Props. of $\otimes$,

$\propto |\boldsymbol{\Sigma}_{spp}|^{-(\nu+n+K+1)/2} \exp\left(-\frac{1}{2}\boldsymbol{\Delta}'\boldsymbol{\Delta}\boldsymbol{\Sigma}_{spp}^{-1}\right) \exp\left(-\frac{1}{2}\mathrm{tr}(\boldsymbol{S}\boldsymbol{\Sigma}_{spp}^{-1})\right)$, Prop. of vec

$\propto |\boldsymbol{\Sigma}_{spp}|^{-(\nu+n+K+1)/2} \exp\left(-\frac{1}{2}\mathrm{tr}(\boldsymbol{\Delta}'\boldsymbol{\Delta}\boldsymbol{\Sigma}_{spp}^{-1})\right) \exp\left(-\frac{1}{2}\mathrm{tr}(\boldsymbol{S}\boldsymbol{\Sigma}_{spp}^{-1})\right)$, Prop. of trace

$\propto |\boldsymbol{\Sigma}_{spp}|^{-(\nu+n+K+1)/2} \exp\left(-\frac{1}{2}\mathrm{tr}([\boldsymbol{\Delta}'\boldsymbol{\Delta} + \boldsymbol{S}]\boldsymbol{\Sigma}_{spp}^{-1})\right)$,

$\implies [\boldsymbol{\Sigma}_{spp}|\cdot] = \text{Inv-Wishart}(\nu + n, \boldsymbol{\Delta}'\boldsymbol{\Delta} + \boldsymbol{S})$.

C ASYMPTOTIC EQUIVALENCE OF POISSON AND LOGISTIC REGRESSION

Consider the models,

$$\begin{aligned} & y_i \sim \text{Bernoulli}(\rho_i),\ \rho_i = 1 - (1-r)^{z_i}, \\ & z_i \sim \text{Bernoulli}(\psi_i),\ \text{logit}(\psi_i) = \boldsymbol{x}_i'\boldsymbol{\beta}, \\ & \text{where } z_i = \mathcal{I}(N_i > 0), \end{aligned} \tag{Model A}$$

$$\begin{aligned} & y_i \sim \text{Bernoulli}(\rho_i),\ \rho_i = 1 - (1-r)^{N_i}, \\ & N_i \sim \text{Pois}(\lambda_i),\ \log(\lambda_i) = \boldsymbol{x}_i'\boldsymbol{\beta}. \end{aligned} \tag{Model B}$$

$$\begin{aligned} \ell_A(\boldsymbol{\beta}|\boldsymbol{Y}) &= \sum_{i=1}^{n}\left(y_i \log\left(\frac{\rho_i}{1-\rho_i}\right) + \log(1-\rho_i) + z_i \log\left(\frac{\psi_i}{1-\psi_i}\right) + \log(1-\psi_i)\right) \\ &= \sum_{z_i=1}\left(y_i \log\left(\frac{r}{1-r}\right) + \log(1-r) + \log\left(\frac{\psi_i}{1-\psi_i}\right) + \log(1-\psi_i)\right) + \\ & \sum_{z_i=0} \log(1-\psi_i). \end{aligned}$$

We denote $n_0 = n - \sum_{i=1}^n z_i$ and let $n_0 \longrightarrow \infty$. Then

$$\ell_A(\boldsymbol{\beta}|\boldsymbol{Y}) \approx \sum_{z_i=0} \log(1-\psi_i) \approx -\sum_{z_i=0} \psi_i,$$

where the approximation follows from the Taylor series expansion of $\log(1-x)$ when x is small. Next, observe

$$\begin{aligned}\ell_B(\boldsymbol{\beta}|\boldsymbol{Y}) &= \sum_{i=1}^n \left(y_i \log\left(\frac{\rho_i}{1-\rho_i}\right) + \log(1-\rho_i) + N_i \log(\lambda_i) - \lambda_i \right) \\ &= \sum_{N_i>0} \left(y_i \log\left(\frac{\rho_i}{1-\rho_i}\right) + \log(1-\rho_i) + N_i \log(\lambda_i) - \lambda_i \right) + \\ &\sum_{N_i=0} -\lambda_i\end{aligned}$$

Again, let $n_0 \longrightarrow \infty$. Then

$$\ell_B(\boldsymbol{\beta}|\boldsymbol{Y}) \approx -\sum_{N_i=0} \lambda_i \approx -\sum_{z_i=0} \psi_i,$$

for ψ_i small. Hence, the likelihoods are approximately equal for $n_0 \longrightarrow \infty$, and inference on $\boldsymbol{\beta}$ will be similar for models A and B.

D HABITAT COVARIATES

Name	Discription
Aspen	Percent of canopy cover that is Aspen
Bare	Percent bare ground of all material < 0.25 m in height
Canopy	Percent canopy cover (all species pooled)
DeadDown	Percent of ground cover due to dead and down trees
ELEV	Elevation of site (meters)
Habitat	Binary indicator of whether site was lodgepole pine or spruce-fir dominated
ShrubHt	Shrub height (meters)
TWIP	Topographic wetness index plus an index of soil moisture based on slope, basin characteristics, and solar radiation
TPI	Topographic position index of concavity or convexity to indicate position along a slope from valley to ridge top
UCCover	Percent of shrub cover due to coniferous species
UDCover	Percent of shrub cover due to deciduous species
WILD	Binary indicator of whether site was located in a federally designated wilderness or not

For details on how habitat covariates were collected, see Ivan et al. (2018).

E SPECIES DESIGN MATRICES

**Species**	**Intensity**	**Detection**
American Marten	Bare, WILD	QUAD
Black Bear	TWIP, ELEV, WILD	LIN
Chipmunk	Canopy, TWIP, TPI	LIN
Coyote	ShrubHt, Bare, TWIP, TPI	CONT
Elk	UCCover, Bare, TWIP, TPI	QUAD
Golden-mantled Ground Squirrel	Aspen, Bare	LIN
Moose	Aspen, ShrubHt, TWIP, ELEV	QUAD
Mule Deer	UDCover, DeadDown, Bare	CONT
Porcupine	Habitat, Bare, WILD	LIN
Red Fox	Aspen, DeadDown, TPI, WILD	LIN
Red Squirrel	Habitat, Bare, WILD	LIN
Snowshoe Hare	Habitat, Aspen, Bare	CONT
Yellow-bellied Marmot	WILD	CONT

In addition to the covariates shown in the table, each design matrix in the intensity model also included a species specific intercept as well as the bark beetle covariates (severity, YSO1, and YSO2). We specified the following for our detection model:

$$\text{logit}(r_j) = \begin{cases} \alpha_0 & ,\text{CONT} \\ \alpha_0 + \alpha_1 j & ,\text{LIN} \\ \alpha_0 + \alpha_1 j + \alpha_2 j^2 & ,\text{QUAD} \end{cases}.$$

We centered the occasions to have mean 0 in the linear model and used orthogonal polynomial basis functions for the quadratic model.
